# Prostate cancer across four countries in the Middle East: a multi-centre, observational, retrospective and prognostic study

**DOI:** 10.3332/ecancer.2024.1695

**Published:** 2024-04-16

**Authors:** Fadi El-Karak, Ali Shamseddine, Ayman Omar, Imene Haddad, Mahmoud Abdelgawad, Manwar Al Naqqash, Mohammad Ali Kaddour, Mohamed Sharaf, Ehab Abdo

**Affiliations:** 1 Hematology and Medical Oncology Department, Hotel Dieu de France University Hospital, Beirut, Lebanon; 2 Clinical Medicine, American University of Beirut, Beirut, Lebanon; 3 Clinical Oncology and Nuclear Medicine Department, Suez Canal University, Ismailia, Egypt; 4 Oncology Department, King Faisal Specialist Hospital and Research Center, Faculty of Medicine, Al Faisal University, Riyadh, Saudi Arabia; 5 Janssen, Issy les Moulineaux, France; 6 Johnson & Johnson, Middle East, Dubai, United Arab Emirates; 7 College of Medicine, University of Baghdad, Baghdad, Iraq; 8 Johnson & Johnson, Middle East FZ-LL, Lebanon; 9 Janssen MEA, Dubai, United Arab Emirates; 10 Cancer Control Center, Kuwait; a https://orcid.org/0000-0002-9266-591X

**Keywords:** Middle East, prostate cancer, patient characteristics, disease progression, treatment patterns

## Abstract

Prostate cancer (PC) is the second most prevalent cancer in males, with a steadily increasing incidence in the Middle East (ME). The aim of this study was to capture real-world data on the characteristics, disease progression, and treatment patterns among PC patients in the ME. This was a retrospective, observational, multi-centre study conducted across ten hospitals/research centers in Lebanon, Kingdom of Saudi Arabia, Iraq and Kuwait. Data were abstracted from medical records of 615 male patients who were diagnosed with PC between January 2012 and the site initiation date (December 2018-May 2019) and received at least one PC treatment/intervention. The observation period ranged between 84 and 88 months. Data were collected on demographics, clinical characteristics, time to progression to the subsequent clinical state or therapy (progression from localised/locally advanced PC to castration and to metastatic PC (metastatic castration-sensitive PC (mCSPC) or metastatic castration-resistant PC (mCRPC)), progression from mCSPC to mCRPC, and mCRPC patients’ progression to first subsequent line of therapy), treatment patterns, and mortality. Most patients had localised/locally advanced PC (57.7%), followed by mCSPC (37.4%), and mCRPC (4.1%) at the time of inclusion in the study. Most patients were at tumours, nodes and metastases (TNM) stage IIIa (40.1%) or TNM stage IVb (27.8%) at study entry. Median time to metastatic disease, castration-resistance and next line therapy was 84 months (95% CI: 68–84), 41 months (95% CI: 30–56) and 7 months (95% CI: 0–41), respectively. The mortality rate was 3.6%. Disease progression was most common among patients with mCSPC (35.1%) or mCRPC (14.8%), and treatment discontinuation was most common among patients with mCRPC (36.6% treatments discontinued). The results show that most patients were at an advanced TNM stage at study entry, suggestive of a lack of awareness regarding PC. Disease progression was most common among patients with metastatic disease, reflecting the challenge of treating metastatic disease and highlighting the need for novel treatments.

## Introduction

Prostate cancer (PC) is the second most prevalent malignancy in men after lung cancer, accounting for 396,773 deaths (4.1% of all cancer-related deaths) and 1,466,718 new cases (7.3% of all new cancer cases) worldwide in 2022 [1, 2]. A 79.1% increase in PC incidence is predicted by 2040 [1, 3]. The age-standardised incidence rate (ASIR) in the Middle East (ME) region was 10.50 per 100,000 person-years (PY) in 2020, compared with 21.50 per 100,000 PY in Europe and North America [4]. However, PC incidence in the Middle East and North Africa (MENA) is steadily increasing, which may be explained by acculturation and lifestyle modifications [5]. In the ME countries of interest in this study – Lebanon, Kingdom of Saudi Arabia (KSA), Iraq, and Kuwait, the reported ASIRs are 73.8, 23.4, 19.9 and 31.2 per 100,000 PY, respectively, and age-standardised mortality rates are 25.7, 9.0, 11.6 and 10.2 per 100,000 PY, respectively [6].

Several treatment modalities, such as radical prostatectomy [7], radiotherapy [7], brachytherapy [8], and androgen deprivation therapy (ADT) [9], have been established for patients with PC based on severity and risk. Radical prostatectomy and/or radiotherapy are the primary treatment options for localised/locally advanced PC [7]. Robotic or laparoscopic prostatectomy is often preferred due to better efficacy and fewer side effects compared to conventional prostatectomy [10]. ADT is often combined with radiotherapy for high-risk patients to minimise systemic side effects and loss of sexual function [9]. However, ADT is the primary treatment option for castration-sensitive PC patients (metastatic or non-metastatic) [11], whereas castration-resistant PC (CRPC) patients require androgen biosynthesis inhibitors [12] or anti-androgens [13–15], alone (for both metastatic or non-metastatic patients) or in combination with cytotoxic agents (for metastatic patients) [16], to improve survival.

The healthcare community and policymakers require real-world data on patients’ characteristics and outcomes to support clinical decisions and to inform clinical practice guidelines. Compared to a randomised clinical trial (RCT), which includes patients who meet specific criteria, a well-designed real-world study covers a broad-spectrum of patients in routine practice [17]. The majority of real-world metastatic or non-metastatic CRPC patients are elderly with comorbidities, such as cardiovascular disease, hypertension, and diabetes mellitus, who are often under-represented in RCTs [12]. The ME region has national cancer registries, where incidence and/or mortality related to different types of cancer (including PC) are reported [18]. However, registries specific to PC are not currently available in the region, and only a limited number of real-world studies on PC have been conducted, hence there are scarce data on patient characteristics, disease progression, and treatment patterns. Thus, the aim of this study was to capture real-world data on patients’ characteristics, clinical states (localised/locally advanced PC, non-metastatic castration-resistant PC (nmCRPC), metastatic castration-sensitive PC (mCSPC), metastatic castration‑resistant PC (mCRPC)), disease progression, and treatment patterns (treatment type, duration of treatment, time to treatment failure) for PC in routine clinical practice in MENA region.

## Patients and methods

### Study design

This was a retrospective observational chart review study, conducted among patients diagnosed with PC, at ten hospitals/research centres across Lebanon, KSA, Iraq and Kuwait. Site selection was based on a range of factors (including geographic location, patient population size, hospital infrastructure quality and PC prevalence) aimed at enhancing patient sample representativeness. Data were retrospectively abstracted from medical records of patients who were diagnosed/presented with PC at the participating sites between 01 January 2012 and the respective dates of site initiation. As each site had a different initiation date, ranging from 27 December 2018 to 09 May 2019, the observation period accordingly ranged between approximately 84–88 months. As this was a descriptive study, a formal sample size was not calculated. Based on feasibility assessment prior to study initiation, a minimum sample size of 500 patients was targeted. Data were entered into electronic case report forms (eCRF) by trained research staff. Informed consent was not required as per national regulations for retrospective studies. The study protocol (available upon request) was submitted and approved by the corresponding Ethics Committees of each participating hospital prior to any study-related activity.

### Study population

Male patients, who were diagnosed and/or presented with confirmed prostate adenocarcinoma and received at least one prostate-specific treatment or intervention (including surgery and radiotherapy), were eligible for study inclusion. Patients whose treatments could not be verified from health records, and/or received PC-specific treatment as part of a clinical trial were excluded.

### Variables

Patient demographics (age and ethnicity), family history of cancer/PC, clinical states, and clinical characteristics (comorbidities, Eastern Cooperative Oncology Group (ECOG) score, histological PC type, tumours, nodes and metastases (TNM) stage, Gleason score, risk group, and baseline prostate-specific antigen (PSA) level) were collected at study entry. The clinical states were defined as localised/locally advanced PC, mCSPC, nmCRPC and mCRPC. Disease progression to subsequent clinical state, treatment patterns (treatment type, duration and time to treatment failure), and survival were documented over the observation period.

### Endpoints

Study endpoints were: (i) Disease course characterisation (time to progression to subsequent clinical state or therapy): (a) For localised/locally advanced PC patients – time to detection of metastatic disease; (b) For nmCRPC patients – time to detection of metastatic disease; (c) For mCSPC patients – time to conversion to castration-resistance, (d) For mCRPC patients – time to first subsequent line of therapy; (ii) Overall mortality rate and time to death; (iii) Duration of PC treatment by clinical state; and (iv) Time to treatment failure (for all treatments and by treatment type).

### Statistical analysis

Statistical analyses were performed using statistical analysis system (SAS) software, version 9.2 (SAS Institute, Cary, NC). Descriptive statistics for continuous variables were summarised by mean with standard deviation (SD); categorical variables were summarised as frequency (*n*) and percentage (%).

Time-to-event endpoints were analysed using Kaplan–Meier survival plots. For all time-to-event analyses, patients who had not experienced the event of interest at the time of study end date were censored. Median survival times with 95% confidence intervals (95% CIs) were calculated.

## Results

### Patient disposition

A total of 10 sites participated in this study (Lebanon: 4 sites, KSA: 3 sites, Iraq: 2 sites, and Kuwait: 1 site). Among a total of 615 eligible patients, the majority were from Lebanon (*n* = 254, 41.3%), followed by KSA (*n* = 147, 23.9%), Kuwait (*n* = 120, 19.5%) and Iraq (*n* = 94, 15.3%).

### Patient demographics, clinical state & comorbidities

Table 1 shows patient demographics and comorbidities at study entry. The majority of patients had localised/locally advanced PC (*n* = 355, 57.7%), followed by mCSPC (*n* = 230, 37.4%), and mCRPC (25, 4.1%); none had nmCRPC (*n* = 0, 0.0%). Overall, the mean (SD) age at study entry was 68.2 (9.3) years. The majority of patients were Caucasian (*n* = 563, 91.5%), and did not report having a family history of cancer (*n* = 448, 90.0%) or PC (*n* = 473, 94.8%). Hypertension (*n* = 275, 67.1%), diabetes (*n* = 189, 46.0%), renal impairment (*n* = 35, 8.5%), and ischemic heart disease (*n* = 24, 5.9%) were the most common comorbidities.

### Patient clinical characteristics

Table 2 shows the clinical characteristics of patients at study entry. Overall, most patients had an ECOG score of either 1 (*n* = 157, 51.6%) or 0 (*n* = 96, 31.6%). An ECOG score of 1 was notably higher among mCRPC (*n* = 12, 75.0%) and mCSPC patients (*n* = 81, 57.9%) versus localised/locally advanced PC patients (*n* = 64, 43.2%). The most common histological PC types were acinar adenocarcinoma (*n* = 279, 89.1%), and ductal adenocarcinoma (*n* = 33, 10.5%). The proportion of patients with ductal adenocarcinoma was notably higher among mCRPC (*n* = 4, 30.8%) and mCSPC patients (*n* = 20, 16.7%) versus localised/locally advanced PC patients (*n* = 9, 5.1%). Overall, most patients were at TNM stage IIIa (*n* = 85, 40.1%) or TNM stage IVb (*n* = 59, 27.8%) at study entry. mCRPC or mCSPC patients were mainly at TNM stage IVb (*n* = 8, 88.9% and *n* = 48, 96.0%, respectively) at study entry, and localised/locally advanced PC patients were mainly at TNM stage IIIa (*n* = 84, 54.9%) or TNM stage IIIb (*n* = 41, 26.8%) at study entry. Overall, most patients had either a Gleason score group of 1 (*n* = 273, 44.4%) or 2/3 (*n* = 133, 21.6%). The proportion of patients with Gleason score group 5 was highest among mCRPC (*n* = 7, 28.0%) or mCSPC patients (*n* = 61, 26.5%) compared with localised/locally advanced PC (*n* = 18, 5.1%).

Overall, most patients had a PSA test recorded at study entry (*n* = 536, 87.2%), and the mean (SD) PSA was 89.3 (521.9) ng/mL. Mean PSA values differed significantly by clinical state (mCSPC: 188.2 (812.7) ng/mL; mCRPC: 99.5 (144.6) ng/mL; localised/locally advanced PC: 17.2 (56.8) ng/mL) (*p=.0012*).

### Characterisation of disease course

**Figure 1.**
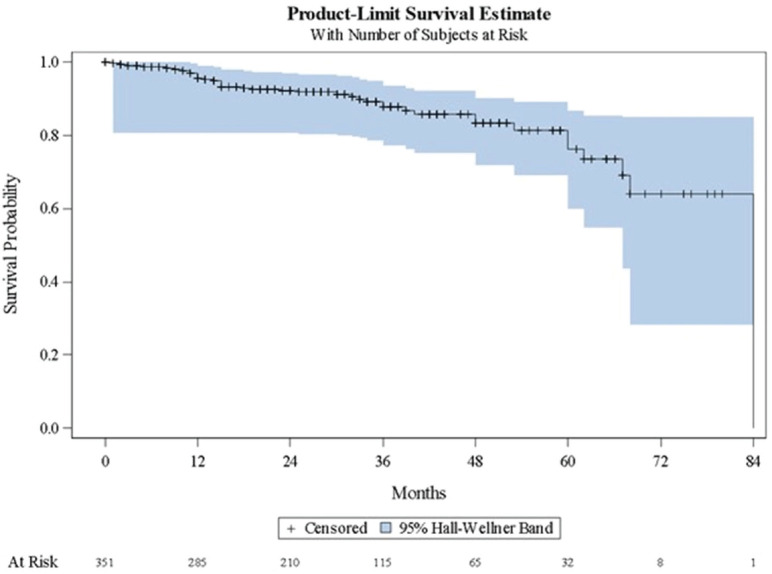
Kaplan–Meier curve describing time to detection of metastatic disease, either mCSPC or mCRPC (localised/locally advanced disease). mCRPC = metastatic castration-resistant prostate cancer; mCSPC = metastatic castration-sensitive prostate cancer.

**Figure 2.**
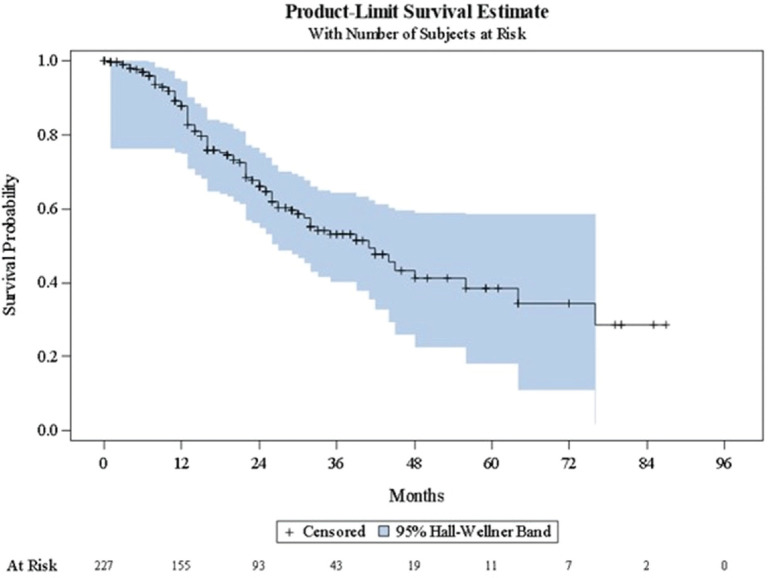
Kaplan–Meier curve describing time to conversion to castration-resistance (mCSPC). mCSPC = metastatic castration-sensitive prostate cancer.

**Figure 3.**
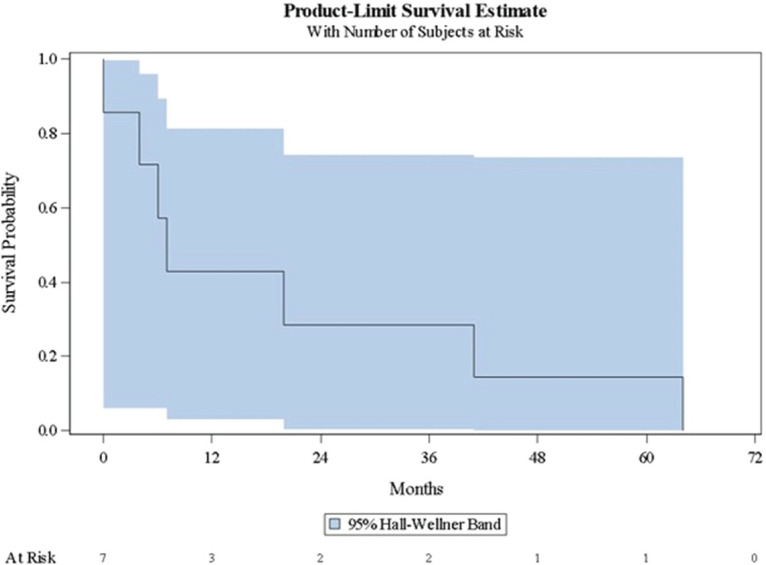
Kaplan–Meier curve describing time to first subsequent line of therapy (mCRPC). *Time to first subsequent line of therapy (mCRPC): either chemotherapy, immunotherapy or bone-targeting drugs. mCRPC = metastatic castration-resistant prostate cancer.

Table 3 shows a summary of the Kaplan-Meier estimates characterising disease progression to subsequent clinical state. Figures 1–3 present the corresponding Kaplan-Meier curves. Among patients with localised/locally advanced PC at study entry, 41 (11.7%) patients advanced to metastatic disease, either mCSPC or mCRPC, during the observation period; median time to metastatic disease was 84 months (95% CI: 68–84). Among patients with mCSPC at study entry, 80 (35.2%) patients converted to castration-resistance during the observation period; median time to castration-resistance was 41 months (95% CI: 30–56). Among patients with mCRPC at study entry, time to first subsequent line of therapy (either chemotherapy, immunotherapy or bone-targeting drugs) following their first line of therapy was assessed; the median time to the first subsequent line of therapy was 7 months (95% CI: 0–41). As there were no patients with nmCRPC at study entry, it was not possible to assess the time to detection of metastatic disease among these patients.

### Overall survival (OS)

**Figure 4.**
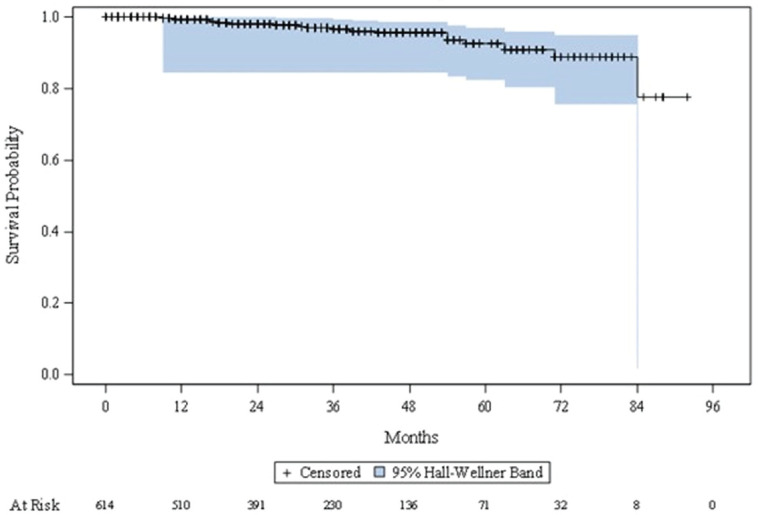
Kaplan–Meier curve describing time to death.

Figure 4 shows the Kaplan-Meier curve of OS. Among 614 patients, 22 (3.6%) patients died. Due to the small number of events, Kaplan–Meier estimates could not be calculated. The Kaplan–Meier curve shows minimal decline over the study observation period. At 84 months from the time of study entry, around 20% of patients had died; after this, the curve plateaus to the end of the observation period.

### Treatment patterns

#### Treatment types

Table 4 shows treatments used among patients by clinical state. Among localised/locally advanced PC patients, a total of 11 specified treatments were used; common treatments were luteinising hormone-releasing hormone (LHRH) agonist (*n* = 174, 26.5%), external-beam radiation therapy (*n* = 151, 23.0%), anti-androgens (*n* = 92, 14.0%), and radical prostatectomy (robotic) (*n* = 83, 12.7%). Among nmCRPC patients, a total of five specified treatments were used: anti-androgens (*n* = 4, 44.4%), external-beam radiation therapy (*n* = 2, 22.2%), radical prostatectomy (open surgery) (*n* = 1, 11.1%), radical prostatectomy (robotic) (*n* = 1, 11.1%), and chemotherapy (*n* = 1; 11.1%). Among mCRPC patients, a total of 11 specified treatments were used; common treatments were LHRH agonist (*n* = 172, 29.6%), anti-androgens (*n* = 135, 23.2%), combined androgen blockade (*n* = 77, 13.3%), and chemotherapy (*n* = 51, 8.8%). Among mCSPC patients, a total of 11 specified treatments were used; common treatments were anti-androgens (*n* = 100, 30.5%), chemotherapy (*n* = 94, 28.7%), bone-targeting drugs (*n* = 45, 13.7%), and corticosteroids (*n* = 26, 7.9%). Chemical castrations (ranging from *n* = 517 (86.2%) among mCRPC patients to *n* = 6 (66.7%) among nmCRPC patients) occurred more frequently compared with surgical castrations (ranging from *n* = 40 (7.7%)) among mCRPC patients to *n* = 0 (0.0%) among nmCRPC patients). In addition, treatment failure (disease progression) was most common among patients with metastatic disease (either mCSPC (35.1%) or mCRPC (14.8%)).

### Treatment duration

**Figure 5.**
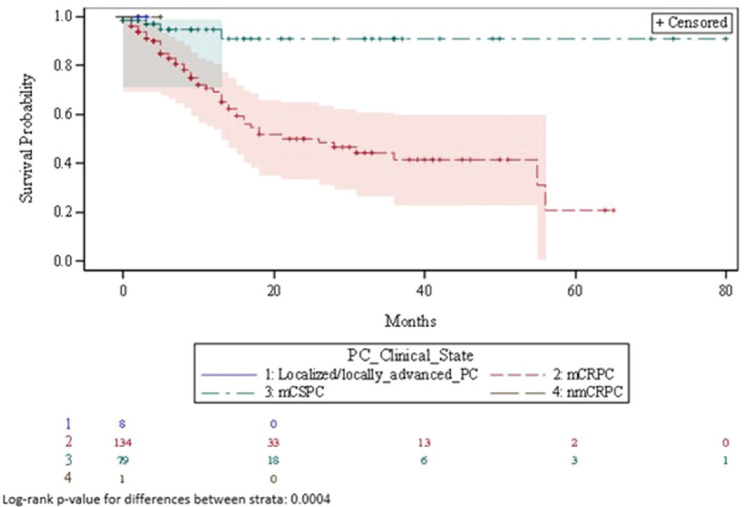
Kaplan-Meier curve describing duration of treatment with PC drugs. mCRPC = metastatic castration-resistant prostate cancer; mCSPC = metastatic castration-sensitive prostate cancer; nmCRPC = non-metastatic castration-resistant prostate cancer; PC = prostate cancer.

Table 5 shows a summary of Kaplan-Meier estimates relating to the duration of treatment with PC drugs (chemotherapy, immunotherapy and bone-targeting drugs). Figure 5 illustrates the corresponding Kaplan-Meier curve. Overall, among all instances of treatment use for chemotherapy, immunotherapy, and bone-targeting drugs, 53 (23.9%) treatments were discontinued during the observation period. Median time to treatment discontinuation was 55 months (95% CI: 28- not computable (NC)). Treatment discontinuation was more common among mCRPC patients (49 (36.6%) treatments discontinued) compared to mCSPC patients (4 (5.1%) treatments discontinued); treatment discontinuation of these drugs did not occur among localised/locally advanced PC or nmCRPC patients. Treatment duration significantly differed by clinical state (*p=.0004*). Overall, the median time to treatment failure was 79 months (95% CI: 22-NC) (Table 6).

## Discussion

This retrospective study provides valuable insights into the characteristics, disease course, and treatment patterns among patients with PC in the real-world clinical setting in the ME. To date, there are scarce published observational data on disease progression and outcomes among patients with PC in the ME, and the current study addresses this gap in research.

The majority of patients in this study had localised/locally advanced PC (57.7%), followed by mCSPC (37.4%), and mCRPC (4.1%) at study entry. The mean age of PC patients (68.2 years) in this study was similar to findings from previous research in the ME (68 years) [19], and globally (66 years) [3]. Hypertension, diabetes, renal impairment, and ischemic heart disease were the main comorbidities observed; these most commonly co-occur with PC [20] and influence treatment selection and patients’ survival [21].

Patients were mainly at TNM stage IIIa (40.1%) or stage IVb (27.8%) at study entry, indicating that this ME study population comprised patients mostly at an advanced TNM cancer stage. This is in line with findings from a recent publication which reported that a high percentage of men present with locally advanced and metastatic PC at diagnosis in the ME [22]. Additionally, a recent ME study, conducted at a large tertiary care centre that receives referrals for PC patients across the region, reported high proportions of patients diagnosed at stage III (Lebanon: 18%, Iraq: 25%, Syria: 33%) and IV (Lebanon: 19%, Iraq: 52%, Syria: 20%) [23]; it is of interest to note the variance in proportions by nationality – however, an important limitation of the study is that the vast majority of patients (71.4%) were Lebanese. The higher proportions of advanced PC stages reported in our study may partly be due to differences in the study populations, as the current study covered a broader range of populations across the ME. A range of factors that may hinder early detection and diagnosis of PC in the ME have been suggested in the literature, including poor knowledge/attitude towards PC examination and screening procedures, mistrust of physicians, fear of diagnosis and testing procedures, and lack of screening programs [24–26]. Physician education with regards to PC screening as well as the importance of counselling patients may support increased uptake of PC screening, and thereby earlier PC diagnosis and improved patient outcomes.

Patients with metastatic disease (mCRPC or mCSPC) at study entry were mainly at TNM stage IVb, and compared to patients with localised/locally advanced PC, were more likely to have a histological PC type of ductal adenocarcinoma, higher Gleason scores, and higher mean PSA values, which are factors associated with poorer PC outcomes [27–30].

It is challenging to directly compare disease progression results of this study with findings from other studies due to differences in study population, patient characteristics, follow-up time, and in some cases, a focus on progression after a single specified treatment. In the current study, 11.7% patients progressed to either mCSPC or mCRPC from localised/locally advanced PC, with a median time of 84 months. Previous studies have reported a wide range of metastatic progression rates (3.4%–21.0%) [31–33]. Among patients diagnosed with mCSPC, 35.2% became castration-resistant, and the median time to castration-resistance was 41 months. In the literature, most studies examined time to castration-resistance from the date of initiation with ADT, thus comparability of these findings with available published data is challenging. A retrospective study by US National Cancer Center reported median time for progression to mCRPC of 13.1 months in patients receiving ADT [34]. In this ME study, the use of novel hormone treatments in addition to ADT among patients may be a contributing factor to the observed longer median time to progression to mCRPC. Further in this study, about 20% of patients died at 84 months, which is slightly higher than the results of a previous large US-based study that reported a 15% death rate among PC patients after 84 months of diagnosis [35]. The less favourable outcomes reported in this study compared to other global studies (i.e., reduced median time from localised/locally advanced PC to castration, relatively high rate of progression to metastatic disease, reduced OS at 84 months) may be due to factors specific to the ME region, such as delayed diagnosis, high variation in cancer care, and limited access to specialist multidisciplinary management [22]. Although the sites that participated in this study were large specialist hospitals, delayed referral due to late diagnosis may have contributed to delayed treatment/sub-par management prior to referral to the specialist site, hence poorer outcomes.

The observed treatment patterns in this study are in line with the PC treatment guidelines, which recommend surgery (radical prostatectomy) and/or radiation therapy for patients with localised/locally advanced PC; while ADT therapy, local radiotherapy, chemotherapy, bone-targeting agents, anti-androgens, and corticosteroids are recommended for metastatic disease [36, 37].

Chemotherapy, immunotherapy, and bone-targeting drugs were mainly used by mCRPC or mCSPC patients. Treatment discontinuation of these therapies was most common among mCRPC patients (36.6%), compared to mCSPC patients (5.1%). In addition, disease progression was most common among patients with metastatic disease (either mCSPC (35.1%) or mCRPC (14.8%)). Collectively, these findings reflect the challenge in treating metastatic disease and highlight the need for enhanced disease management. mCRPC, in particular, is a terminal disease despite significant advancements in the development of novel treatment options such as poly ADP-ribose polymerase inhibitors, novel androgen receptor inhibitors, radiopharmaceuticals, and novel immunotherapies (e.g., tumour-associated antigen-directed therapies, immune checkpoint inhibitor therapy) [38].

## Limitations

The findings of this study should be considered in light of the following limitations. First, selection bias needs to be considered because a limited number of sites per country were included in this study, which may limit generalisability of the study results to the general PC population in the MENA region. Second, there was notable missing data on certain clinical characteristics (e.g., ECOG score, histological PC type, TNM stage), and therefore this data needs to be interpreted with caution. Third, specific definitions for each clinical state were not provided in the study protocol/eCRF (clinical state was recorded in the eCRF at the discretion of the investigator and based on their clinical evaluation at each standard of care visit); this may have introduced misclassification bias. Fourth, for some of the Kaplan-Meier analyses, a small number of events impacted the power of the analyses and prevented the computation of estimates. Longer follow-up times should be considered in future studies to capture a greater number of events, thereby increasing the power of the analyses.

## Conclusion

This observational study captured real-world data on the characteristics, disease course, and treatment patterns among patients with PC in the ME. Most patients were at an advanced TNM stage at study entry, suggestive of limited awareness regarding PC and highlighting a need for PC screening programs in the region. Disease progression was most common among patients with metastatic disease (mCSPC or mCRPC), and treatment discontinuation of chemotherapy, immunotherapy, and/or bone-targeting drugs was most common among mCRPC patients. These results reflect the challenge in treating metastatic disease, underscoring the need for novel treatments to improve outcomes and alleviate the burden associated with PC.

## Conflicts of interest

Fadi El-Karak, Ali Shamseddine, Ayman Omar, Manwar Al Naqqash and Ehab Abdo have no conflicts of interest. Imene Haddad, Mahmoud Abdelgawad, Mohammad Ali Kaddour and Mohamed Sharaf are employees of Johnson & Johnson Medical Affairs Department and were also part of the medical team supporting the work on the study and the manuscript. Imene Haddad is an EMEA Medical Program Lead, Mahmoud Abdelgawad is Gulf Medical Lead, Mohammad Ali Kaddour is a NEMA Medical Advisor, and Mohamed Sharaf is an EMEA Senior Medical Advisor. Mohamed Sharaf is a shareholder of Johnson & Johnson.

## Funding

This work was funded by Janssen Pharmaceuticals, Inc.

## Figures and Tables

**Table 1.** Patient demographics and comorbidities at study entry.

	Overall study population (*N* = 615)	Localised/locally advanced PC (*N* = 355)	mCRPC (*N* = 25)	mCSPC (*N* = 230)
*n* (%)				
Age (years)				
Mean (SD)	68.2 ± 9.3	67.0 ± 8.9	67.8 ± 10.2	70.0 ± 9.5
Ethnicity				
Caucasian	563 (91.5)	335 (94.4)	22 (88.0)	201 (87.4)
Black	3 (0.5)	2 (0.6)	0 (0.0)	1 (0.4)
Native American	1 (0.2)	1 (0.3)	0 (0.0)	0 (0.0)
Asian/Pacific Islander	12 (2.0)	7 (2.0)	0 (0.0)	5 (2.2)
Other	34 (5.5)	10 (2.8)	3 (12.0)	21 (9.1)
Unknown	2 (0.3)	0 (0.0)	0 (0.0)	2 (0.9)
Family history of any cancer	498 (81.0)	287 (80.8)	23 (92.0)	187 (81.3)
Yes	50 (10.0)	29 (10.1)	3 (13.0)	18 (9.6)
No	448 (90.0)	258 (89.9)	20 (87.0)	169 (90.4)
Family history of PC	499 (81.1)	286 (80.6)	23 (92.0)	189 (82.2)
Yes	26 (5.2)	16 (5.6)	1 (4.3)	9 (4.8)
No	473 (94.8)	270 (94.4)	22 (95.7)	180 (95.2)
Comorbidities				
Diabetes	411 (66.8)	254 (71.5)	8 (32.0)	146 (63.5)
Yes	189 (46.0)	110 (43.3)	5 (62.5)	74 (50.7)
No	222 (54.0)	144 (56.7)	3 (37.5)	72 (49.3)
Liver disease	412 (67.0)	255 (71.8)	8 (32.0)	146 (63.5)
Yes	4 (1.0)	3 (1.2)	0 (0.0)	1 (0.7)
No	408 (99.0)	252 (98.8)	8 (100.0)	145 (99.3)
Renal impairment	411 (66.8)	254 (71.5)	8 (32.0)	146 (63.5)
Yes	35 (8.5)	22 (8.7)	0 (0.0)	13 (8.9)
No	376 (91.5)	232 (91.3)	8 (100.0)	133 (91.1)
Hypertension	410 (66.7)	253 (71.3)	8 (32.0)	146 (63.5)
Yes	275 (67.1)	170 (67.2)	6 (75.0)	97 (66.4)
No	135 (32.9)	83 (32.8)	2 (25.0)	49 (33.6)
Congestive heart failure	410 (66.7)	253 (71.3)	8 (32.0)	146 (63.5)
Yes	19 (4.6)	13 (5.1)	0 (0.0)	6 (4.1)
No	391 (95.4)	240 (94.9)	8 (100.0)	140 (95.9)
COPD	411 (66.8)	254 (71.5)	8 (32.0)	146 (63.5)
Yes	8 (1.9)	6 (2.4)	0 (0.0)	2 (1.4)
No	403 (98.1)	248 (97.6)	8 (100.0)	144 (98.6)
Stroke	411 (66.8)	254 (71.5)	8 (32.0)	146 (63.5)
Yes	6 (1.5)	5 (2.0)	0 (0.0)	1 (0.7)
No	405 (98.5)	249 (98.0)	8 (100.0)	145 (99.3)
PVD	408 (66.3)	253 (71.3)	8 (32.0)	145 (63.0)
Yes	3 (0.7)	2 (0.8)	0 (0.0)	1 (0.7)
No	405 (99.3)	251 (99.2)	8 (100.0)	144 (99.3)
Ischemic heart disease	410 (66.7)	253 (71.3)	8 (32.0)	146 (63.5)
Yes	24 (5.9)	12 (4.7)	0 (0.0)	12 (8.2)
No	386 (94.1)	241 (95.3)	8 (100.0)	134 (91.8)

mCRPC = metastatic castration-resistant prostate cancer; mCSPC = metastatic castration-sensitive prostate cancer; n = number of patients; SD = standard deviation; PC = Prostate cancer; COPD = chronic obstructive pulmonary disorder; PVD = peripheral vascular disease

**Table 2.** Patient clinical characteristics at study entry.

	Overall study population (*N* = 615)	Localised/locally advanced PC (*N* = 355)	mCRPC (*N* = 25)	mCSPC (*N* = 230)
ECOG score	304 (49.4)	148 (41.7)	16 (64.0)	140 (60.9)
Score 0	96 (31.6)	64 (43.2)	2 (12.5)	30 (21.4)
Score 1	157 (51.6)	64 (43.2)	12 (75.0)	81 (57.9)
Score 2	40 (13.2)	17 (11.5)	2 (12.5)	21 (15.0)
Score 3	8 (2.6)	3 (2.0)	0 (0.0)	5 (3.6)
Score 4	3 (1.0)	0 (0.0)	0 (0.0)	3 (2.1)
Score 5	0 (0.0)	0 (0.0)	0 (0.0)	0 (0.0)
Histological PC type	313 (50.9)	175 (49.3)	13 (52.0)	120 (52.2)
Acinar adenocarcinoma	279 (89.1)	165 (94.3)	9 (69.2)	100 (83.3)
Ductal adenocarcinoma	33 (10.5)	9 (5.1)	4 (30.8)	20 (16.7)
Transitional cell cancer	1 (0.3)	1 (0.6)	0 (0.0)	0 (0.0)
TNM stage group	212 (34.5)	153 (43.1)	9 (36.0)	50 (21.7)
Stage I	0 (0.0)	0 (0.0)	0 (0.0)	0 (0.0)
Stage IIa	5 (2.4)	5 (3.3)	0 (0.0)	0 (0.0)
Stage IIb/Stage IIc	20 (9.4)	20 (13.1)	0 (0.0)	0 (0.0)
Stage IIIa	85 (40.1)	84 (54.9)	1 (11.1)	0 (0.0)
Stage IIIb	43 (20.3)	41 (26.8)	0 (0.0)	2 (4.0)
Stage IVb	59 (27.8)	3 (2.0)	8 (88.9)	48 (96.0)
Gleason score group	615 (100.0)	355 (100.0)	25 (100.0)	230 (100.0)
Grade 1	273 (44.4)	219 (61.7)	9 (36.0)	44 (19.1)
Grade 2/Grade 3	133 (21.6)	73 (20.6)	5 (20.0)	54 (23.5)
Grade 4	121 (19.7)	45 (12.7)	4 (16.0)	71 (30.9)
Grade 5	88 (14.3)	18 (5.1)	7 (28.0)	61 (26.5)
Risk group	613 (99.7)	355 (100.0)	24 (96.0)	230 (100.0)
Very low	3 (0.5)	3 (0.8)	0 (0.0)	0 (0.0)
Low	27 (4.4)	27 (7.6)	0 (0.0)	0 (0.0)
Favourable intermediate	97 (15.8)	97 (27.3)	0 (0.0)	0 (0.0)
Unfavourable intermediate	38 (6.2)	38 (10.7)	0 (0.0)	0 (0.0)
High	127 (20.7)	124 (34.9)	0 (0.0)	0 (0.0)
Very high	42 (6.9)	41 (11.5)	0 (0.0)	0 (0.0)
Regional	23 (3.8)	23 (6.5)	0 (0.0)	0 (0.0)
Metastatic	256 (41.8)	2 (0.6)	24 (100.0)	230 (100.0)
PSA test performed	536 (87.2)	296 (83.4)	24 (96.0)	214 (93.0)
PSA at baseline (ng/mL)				
Mean (SD)	89.3 ± 521.9	17.2 ± 56.8	99.5 ± 144.6	188.2 ± 812.7

mCRPC = metastatic castration-resistant prostate cancer; mCSPC = metastatic castration-sensitive prostate cancer; n = number of patients PC = Prostate cancer; SD = standard deviation; ECOG = Eastern Cooperative Oncology Group; PSA = prostate-specific antigen; TNM =tumour nodes metastasis

**Table 3.** Summary of the Kaplan-Meier estimates for disease progression.

	Number of patients n (%)	Median (Q1, Q3)	Min, Max	95% Confidence interval	Sum of person time	Events	Censored
Time to detection of metastatic disease (either mCSPC or mCRPC) for localised/locally advanced PC	351 (98.9)	84 (62,84)	(0.84)	(68–84)	1116	41	310
Time to conversion to castration-resistance for mCSPC	227 (98.7)	41 (19,NC*)	(0.87)	(30–56)	1630	80	147
Time to first subsequent line of therapy for mCRPC	7 (28.0)	7 (4,41)	(0.64)	(0–41)	142	7	-

Time-to-event duration is in months. Time to first subsequent line of therapy (mCRPC): either chemotherapy, immunotherapy, or bone-targeting drugs

* A small number of events impacted the power of the Kaplan-Meier analysis and prevented computation of estimates

mCRPC = metastatic castration-resistant prostate cancer; mCSPC = metastatic castration-sensitive prostate cancer; NC = non-computable estimates; Q1 = Quartile 1, Q3 = quartile 3; SD = standard deviation; PC = Prostate cancer

**Table 4.** Summary of treatment among PC patients.

Treatment	Localised/locally advanced PC	nmCRPC	mCRPC	mCSPC	Missing clinical state
	*n* (%)				
Number of treatments	656	9	581	328	20
Type of treatment					
Radical prostatectomy (open surgery)	35 (5.3)	1 (11.1)	1 (0.2)	0 (0.0)	1 (5.0)
Radical prostatectomy (robotic)	83 (12.7)	1 (11.1)	5 (0.9)	1 (0.3)	3 (15.0)
External-beam radiation therapy	151 (23.0)	2 (22.2)	32 (5.5)	12 (3.7)	1 (5.0)
Brachytherapy	4 (0.6)	0 (0.0)	0 (0.0)	0 (0.0)	0 (0.0)
Bilateral orchiectomy	6 (0.9)	0 (0.0)	12 (2.1)	2 (0.6)	2 (10.0)
LHRH agonist	174 (26.5)	0 (0.0)	172 (29.6)	23 (7.0)	6 (30.0)
LHRH antagonists	29 (4.4)	0 (0.0)	28 (4.8)	1 (0.3)	0 (0.0)
Anti-androgens	92 (14.0)	4 (44.4)	135 (23.2)	100 (30.5)	6 (30.0)
Combined androgen blockade	61 (9.3)	0 (0.0)	77 (13.3)	2 (0.6)	0 (0.0)
Chemotherapy	9 (1.4)	1 (11.1)	51 (8.8)	94 (28.7)	0 (0.0)
Immunotherapy	0 (0.0)	0 (0.0)	0 (0.0)	2 (0.6)	0 (0.0)
Bone-targeting drugs	0 (0.0)	0 (0.0)	34 (5.9)	45 (13.7)	0 (0.0)
Corticosteroids	2 (0.3)	0 (0.0)	13 (2.2)	26 (7.9)	0 (0.0)
Others	10 (1.5)	0 (0.0)	20 (3.4)	20 (6.1)	0 (0.0)
Castration	707	9	600	334	25
Chemical	477 (67.5)	6 (66.7)	517 (86.2)	275 (82.3)	18 (72.0)
Surgical	40 (5.7)	0 (0.0)	46 (7.7)	40 (12.0)	4 (16.0)
Treatment failure (disease progression)	656	9	581	328	20
Yes	37 (5.6)	1 (11.1)	86 (14.8)	115 (35.1)	3 (15.0)
No	606 (92.4)	7 (77.8)	451 (77.6)	193 (58.8)	15 (75.0)

mCRPC = metastatic castration-resistant prostate cancer; mCSPC = metastatic castration-sensitive prostate cancer; n = number of treatments; nmCRPC = non-metastatic castration-resistant prostate cancer; LHRH = luteinising hormone-releasing hormone

**Table 5.** Summary of Kaplan-Meier estimates for duration of treatment with PC drugs.

Strata	Number of treatments n (%)	Median (Q1, Q3)	Min, Max	95% CI	Sum of person time	Events	Censored
Duration of treatment with PC drugs	222 (94.1)	55 (13, NC*)	(0,80)	(28, NC*)	615	53	169
Localised/locally advanced PC	8 (3.6)	NC*	(0,3)	NC*	0	0	8
nmCRPC	1 (0.5)	NC*	(0,5)	NC*	0	0	1
mCRPC	134 (60.4)	26 (9,56)	(0,65)	(15,56)	594	49	85
mCSPC	79 (35.6)	NC*	(0,80)	NC*	21	4	75

Time-to-event duration is in months. Duration of three treatments (chemotherapy, immunotherapy and bone-targeting drugs) is assessed. Clinical states and duration of treatment with PC drugs are number of instances and not the number of patients (a single patient can receive one or more of any of the three treatments). Log-rank *p*-value for differences between strata: 0.0004

* A small number of events impacted the power of the Kaplan-Meier analysis and prevented computation of estimates

mCRPC = metastatic castration-resistant prostate cancer; mCSPC = metastatic castration-sensitive prostate cancer; n = number of treatments; NC= non-computable estimates; nmCRPC= non-metastatic castration-resistant prostate cancer

**Table 6.** Summary of Kaplan-Meier estimates for time to failure for each treatment type.

Strata	Number of treatments *n* (%)	Median (Q1, Q3)	Min, Max	95% CI	Sum of person time	Events	Censored
Treatment type	1,488 (93.4)	79 (22,NC*)	(0.83)	(57, NC*)	2,567	228	1,260
Radical prostatectomy (open surgery)	36 (2.4)	NC*	(0.74)	NC*	0	3	33
Radical prostatectomy (robotic)	92 (6.2)	NC*	(0.54)	NC*	0	0	92
External-beam radiation therapy	177 (11.9)	NC* (41,NC*)	(0.65)	(20, NC*)	110	5	172
Brachytherapy	3 (0.2)	0 (0, NC*)	(0.47)	(0, NC*)	0	2	1
Bilateral orchiectomy	21 (1.4)	NC*	(0.50)	NC*	0	4	17
LHRH agonist	355 (23.9)	NC* (46, NC*)	(0.83)	(55, NC*)	791	41	314
LHRH antagonists	50 (3.4)	45 (23, NC*)	(0.59)	(34, NC*)	160	10	40
Anti-androgens	310 (20.8)	65 (12,79)	(0.79)	(30,79)	885	71	239
Combined androgen blockade	138 (9.3)	NC* (19, NC*)	(0.54)	(22, NC*)	180	12	126
Chemotherapy	145 (9.7)	8 (4,17)	(0.65)	(6,13)	217	43	102
Immunotherapy	2 (0.1)	NC*	(0.16)	NC*	0	0	2
Bone-targeting drugs	75 (5)	NC* (29, NC*)	(0.80)	(29, NC*)	79	12	63
Corticosteroids	41 (2.8)	9 (3, NC*)	(0.46)	(5, NC*)	59	16	25
Others	43 (2.9)	25 (9, NC*)	(0.46)	(15, NC*)	86	9	34

Time-to-event duration is in months. The denominator for the total number for treatment type is 1,594. The number of instances for each treatment type (and not the number of patients) is presented. Log-rank *p*-value for differences between strata: <0.0001.

* A small number of events impacted the power of the Kaplan-Meier analysis and prevented computation of estimates

CI = confidence interval, LHRH = luteinising hormone-releasing hormone, NC = non-computable estimates
